# GCASSN: a graph convolutional attention synergistic segmentation network for 3D plant point cloud segmentation

**DOI:** 10.3389/fpls.2025.1621934

**Published:** 2025-10-13

**Authors:** Yibo Zou, Haoqiang Wang, Feng Zhang, Yan Ge, Wenjuan Wang, Ming Chen

**Affiliations:** ^1^ College of Information Technology, Shanghai Ocean University, Shanghai, China; ^2^ Key Laboratory of Fisheries Information, Ministry of Agriculture and Rural Affairs, Shanghai, China; ^3^ Bright Food Group Shanghai Chongming Farm Co., Ltd., Shanghai, China

**Keywords:** plant phenotype, 3D plant point cloud, graph convolutional neural network, self-attention mechanism, feature extraction

## Abstract

Plant phenotyping analysis serves as a cornerstone of agricultural research. 3D point clouds greatly improve the problem of overlapping and occlusion of leaves in two-dimensional images and have become a popular field of plant phenotyping research. The realization of faster and more effective plant point cloud segmentation is the basis and key to the subsequent analysis of plant phenotypic parameters. To balance lightweight design and segmentation precision, we propose a Graph Convolutional Attention Synergistic Segmentation Network (GCASSN) specifically for plant point cloud data. The framework mainly comprises (1) Trans-net, which normalizes input point clouds into canonical poses; (2) Graph Convolutional Attention Synergistic Module (GCASM), which integrates graph convolutional networks (GCNs) for local feature extraction and self-attention mechanisms to capture global contextual dependencies. Complementary advantages are realized. On plant 3D point cloud segmentation via the Plant3D and Phone4D datasets, the model achieves state-of-the-art performance with 95.46% mean accuracy and 90.41% mean intersection-over-union (mIoU), surpassing mainstream methods (PointNet, PointNet++, DGCNN, PCT, and Point Transformer). The computational efficiency is competitive, with the inference time and parameter quantity slightly exceeding that of the DGCNN. Without parameter tuning, it attains 85.47% mIoU and 82.9% mean class IoU on ShapeNet, demonstrating strong generalizability. The method proposed in this article can fully extract the local detail features and overall global features of plants, and efficiently and robustly complete the segmentation task of plant point clouds, laying a solid foundation for plant phenotype analysis. The code of the GCASSN can be found in https://github.com/fallovo/GCASSN.git.

## 1 Introduction

The plant phenotype is a collection of physical, physiological, and biochemical characteristics that reflect the structure and function of plant cells, tissues, organs, plants, and populations (Araus and Cairns, 2014). Plant phenomics, as an emerging discipline, focuses on the study of the plant growth process, external expression morphology and internal components of plants (Lai et al., 2021). This study aimed to reveal the patterns of plant growth and development and their interactions with the environment through the collection and analysis of phenotypic data.

Previously, 2D image-based computer vision was an important solution to the plant segmentation problem. However, 2D images suffer from the limitations of insufficient spatial information and image occlusion (Zeng et al., 2023a). In contrast, 3D point clouds can capture the morphological information of plants more comprehensively, which is important for plant phenotyping research. It has now become an important tool for plant phenotyping research (Wang et al., 2023).

In the field of plant 3D point cloud segmentation, there has been significant development from traditional machine learning algorithms to deep learning-based plant 3D point cloud segmentation. Traditional machine learning methods primarily depend on manually designed feature classifiers, where the algorithms have inherent constraints and the feature representation capacity is restricted (Saeed et al., 2023). Deep learning methods are able to learn features automatically, have a great improvement in generalization ability and segmentation effect (Yang et al., 2025).

With the continuous development of deep learning technology and the improvement of hardware computing power, plant point cloud segmentation based on deep learning has become a current research hotspot (Lu et al., 2023). Today, 3D point cloud segmentation algorithms based on deep learning can be divided into three categories:

(1) Point-wise Convolution Methods (e.g., PointNet (Qi et al., 2017a)). These methods process individual points using multi-layer perceptrons (MLPs) to extract local features, with global features aggregated through max pooling. However, it lacks localized feature extraction capability and fails to capture plant details. Xu (Xu et al., 2024) enhanced PointNet++ (Qi et al., 2017b) by optimizing relative feature values of neighboring points and integrating multi-level features. The incorporation of coordinate attention modules and attention pooling improved local feature extraction, boosting tree species classification and crown/trunk segmentation accuracy. Hang (Huang, 2023) proposed a network generating fixed-size point patches, using a PointNet-based feature extractor with residual connections for high-dimensional feature extraction.

(2) Graph Convolution Methods (e.g., RGCNN (Te et al., 2018)). These methods construct graph structures to encode point cloud topology, effectively capturing local geometry with scalability and robustness. However, they require significant memory and struggle with long-range dependencies. Liu (Liu C et al.), proposed the TSNet model to segment tree point clouds by improving the DGCNN (Wang et al., 2019) deep learning network. The feature extraction layer combines graph convolution, ordinary convolution, and recursive gated convolution to train features. Zhong (Zhong, 2022) proposed a GCNN model with dynamic graph reconstruction across layers to extract geometric and semantic features. Accurate segmentation of multiple plants was achieved.

(3) Attention-based Methods (e.g., Point Transformer (Zhao et al., 2021)). These approaches utilize attention mechanisms to model long-range dependencies, demonstrating strong feature extraction capabilities despite high computational costs. Zeng (Zeng et al., 2023b) designed the Multi-Task Segmentation Network (MT-SegNet) model by improving Point Transformer, combined with the Multi-Valued Conditional Random Field (MV-CRF) model to realize stem-leaf semantic segmentation and leaf instance segmentation. Tang (Tang et al., 2024) proposed the Local Global Feature Fusion Segmentation Network (LGF-SegNet) model to better represent the geometric features of the plant point cloud by introducing a dual weighted attention mechanism module and position coding.

Plant point clouds exhibit complex structural characteristics, featuring multi-layered and non-uniform geometric distributions (Miao et al., 2022). Leaves, stems, flowers, and fruits display diverse 3D morphologies, for example leaves may twist or fold, and stems branch or grow in spirals. Plant organs often shade each other, resulting in self-occlusion, for example upper leaves cover lower structures, and overlapping edges cause uneven point cloud density (Nie et al., 2022). The complex nature of plant point clouds poses a challenge to the segmentation task.

An important idea for effectively segmenting plant point clouds is to extract sufficient local-global features (Li and Guo, 2022). Graph convolutional neural networks and attention mechanisms provide excellent solutions. Graph convolution acquires rich local feature information by constructing local graphs with graph structures, but its receptive field is constrained by the neighborhood range of the graph, making it challenging to directly capture global context (Simonovsky and Komodakis, 2017). In comparison, the self-attention mechanism effectively captures comprehensive global contextual information through point-to-point correlation calculations (Park et al., 2023).

Inspired by the collaborative design of graph convolution and attention mechanisms, several studies have emerged: Zhou H’s AdaptConv (Zhou et al., 2021) uses attention mechanisms to adaptively adjust edge weights to optimize graph structures; Wang L’s Generate Adversarial-driven Cross-aware Network (GACNet) (Wang et al., 2019) achieves dynamic focusing on key features in neighborhoods through differentiated attention weight allocation; Chen C introduces an attention aggregation layer to identify core features in neighborhoods, significantly enhancing the robustness of local representations (Chen et al., 2021). All three approaches utilize attention mechanisms to selectively enhance neighborhood features, offering new insights for graph structure learning. However, they all seem to use attention mechanisms to enhance the feature extraction capabilities of graph convolutions. They have not been able to fully leverage the synergistic advantages of graph convolutional neural networks and attention mechanisms.

In order to fully leverage the unique advantages of graph convolutions and attention mechanisms respectively, we integrate the advantages of both graph convolution and attention mechanisms to design the Graph convolutional attention synergism segmentation network (GCASSN). This network effectively captures local geometric structures and detailed information in plant point clouds through graph convolution, while simultaneously integrating global features efficiently using the attention mechanism. This dual approach enables more accurate and efficient segmentation of complex, variable plant point cloud data.

Our main work is as follows:

- The Graph convolutional attention synergism segmentation network(GCASSN) is designed to effectively cope with the irregularity and complexity of plants and realize the efficient segmentation of plant point clouds.
- Graph convolutional attention synergy module(GCASM) is designed to integrate the advantages of graph convolution and attention mechanism, so as to better take into account the local and global feature extraction.
- Trans-net is designed to transform input point clouds into standardized postures, thereby enhancing pose comprehension capability while improving model stability and generalization performance.
- Extensive experimental results show that GCASSN achieves an optimal balance between performance and efficiency.

## 2 Methods

**Figure 1:**
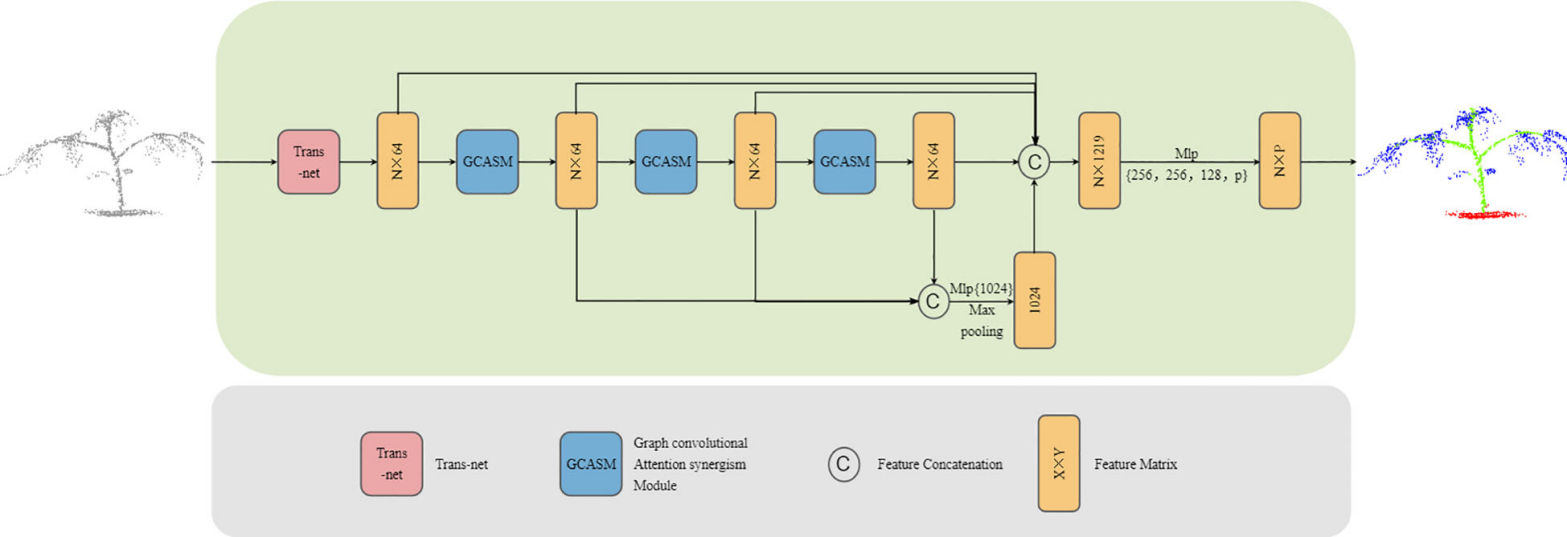
GCASSN architecture.

In order to fully extract the feature information of plants and achieve high-precision segmentation of plant point clouds, we propose an efficient graph convolution attention collaborative segmentation network—GCASSN. The network is mainly composed of Trans-net and GCASM. Trans-net transforms the plant point cloud into a standard pose, highlights the key features of the plant point cloud, and improves the feature comprehension ability of the neural network; GCASM extracts sufficient local-global features with the help of graph convolution and attention mechanism. The GCASSN network architecture is shown in **Figure 1**.

The input of the GCASSN is an N×3 plant point cloud block representing a plant composed of N points, where each point is represented in vector format (i.e., XYZ coordinate information). First, the input point cloud is adjusted to a standard pose through the Trans-net. Second, the standard pose is subjected to feature extraction through the GCASM, where the graph convolution part is used to extract sufficient local features and the attention part performs the global feature collation

After passing through three hierarchical GCASM layers, multi-level features are obtained. Then, the outputs from the three GCASM layers are concatenated and adjusted to 1024 dimensions via MLP to enhance feature representation capability. A max pooling operation is then applied to retain the most salient feature information, producing global features. Subsequently, the standardized pose point cloud, the three-layer GCASM features, and the global features are concatenated. Finally, By progressively mapping the concatenated high-dimensional features to a predefined category space through multiple linear layers and Dropout layers, the final output is an N×P matrix (where N is the number of points and P is the number of target categories).

### 2.1 Trans-net

**Figure 2:**
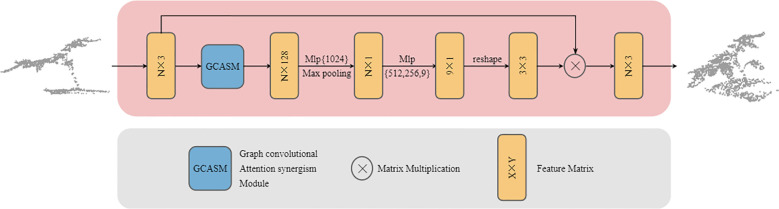
GCASM architecture.

Point cloud data refers to a collection of discrete points in 3D space, characterized by unordered structure and pose diversity. Variations in acquisition devices and viewing angles can lead to significant differences in point cloud representations (Qiao et al., 2025). Directly processing the original point cloud with inherent spatial posture may hinder the neural network from extracting stable features (Guo et al., 2020). We designed Trans-net to enhance the model’s robustness against different plant poses and improve segmentation accuracy. Its structure shown in **Figure 2**.

The input to Trans-Net is an N×3-dimensional plant point cloud patch, consisting of N points with XYZ 3D coordinate information. First, the input data passes through the GCASM module to aggregate both local and global contextual information. Second, the features are adjusted to 1,024 dimensions via MLP (multilayer perceptron) to enhance their representational capacity, while employing max pooling to retain salient features. Subsequently, the channel dimensions are progressively reduced to 512, 256, and finally 9 through stepwise MLP layers, transforming high-dimensional complex features into a more compact and task-specific representation. Finally, the feature is shaped into a 3×3 transformation matrix, which is multiplied by the original input to generate a standard pose aligned point cloud. Trans-net performs end-to-end training with the main network, and its parameters are stored independently. This ensures that the model can continuously adjust the optimal plant posture with subsequent training, thereby improving the segmentation performance of the model.

**Figure 3:**
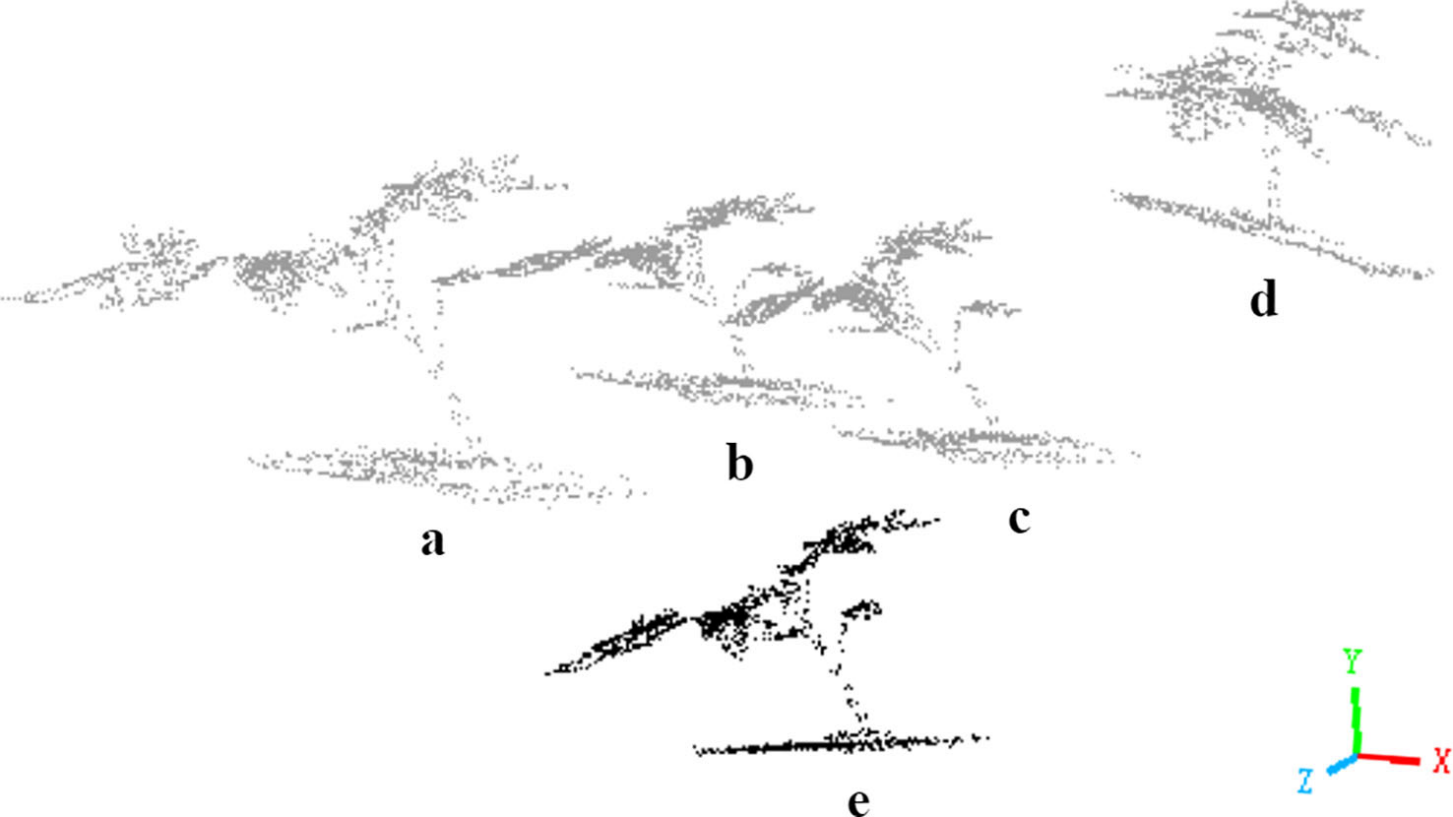
Schematic diagram of attitude transformation in the same coordinate system. Here, **(e)** represents the input point cloud, and **(a-d)** represents the four pose transformations.

By learning a variable transformation matrix that incorporates geometric operations such as rotation, translation, and scaling, Trans-Net aligns input plant point clouds to a standardized pose. As illustrated in **Figure 3**, when an input plant point cloud (**Figure 3e**) passes through Trans-Net, the model iteratively explores and refines the optimal standardized pose for downstream network processing. The four gray plant instances (**Figures 3a–d**) represent four intermediate attempts by the model to optimize the point cloud pose during training. Through this dynamic adjustment mechanism, Trans-Net automatically identifies the most effective transformation, establishing a robust foundation for subsequent feature extraction and segmentation tasks.

Spatial Transformation strengthens plant point cloud processing by addressing pose variations such as leaf tilting and stem bending. This technique standardizes input data for subsequent feature extraction and segmentation, improving geometric accuracy in plant structure representation (Zhang et al., 2017). The process ensures stable network operation and precise outputs while reducing performance loss from pose inconsistencies.

### 2.2 GCASM

As an important part of deep learning, feature extraction greatly affects the performance and application effect of the model (Slimani et al., 2024). For processing plant 3D point cloud data, the core objective of model design is to autonomously learn discriminative and representative features. Plant point clouds contain fine-grained structures (leaf edges, stem surface curvature, and surface textures), as well as macroscopic attributes (plant height, canopy volume, and main stem orientation) (Zhang et al., 2020). The inherent characteristics of this data determine that both local features and global features are equally indispensable.

**Figure 4:**
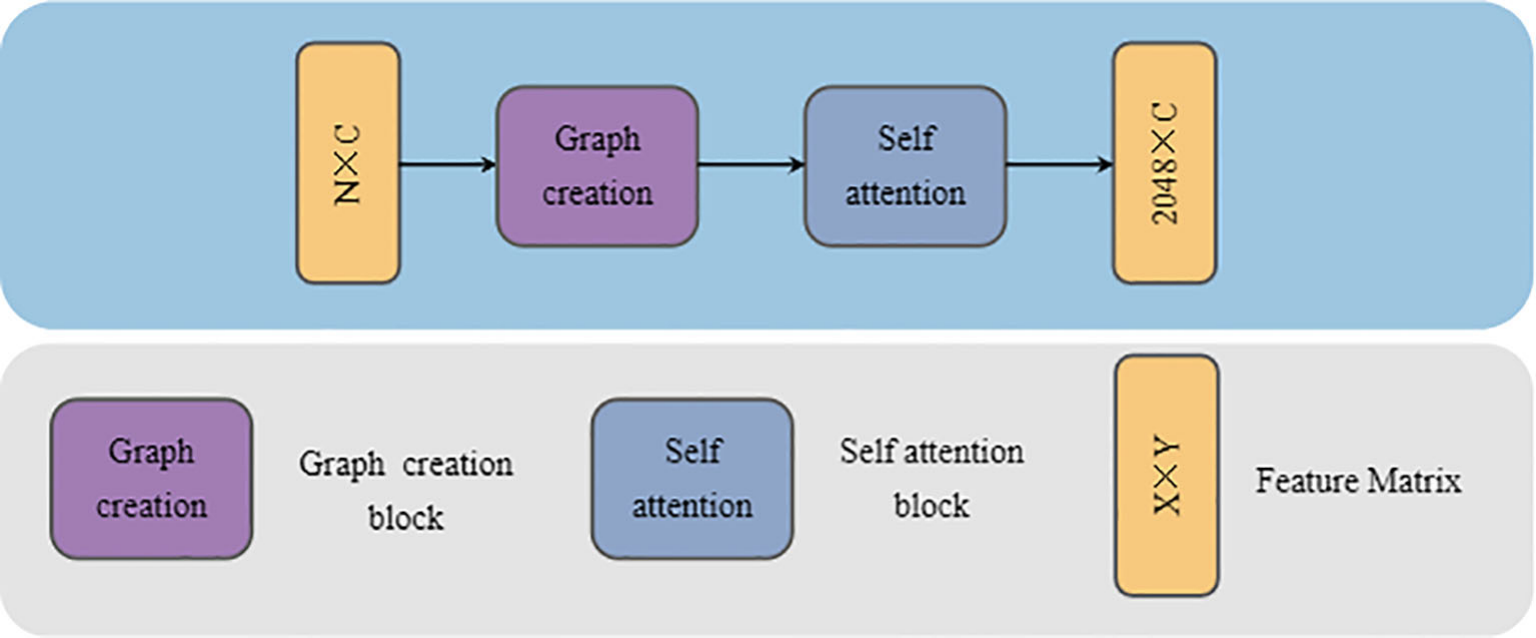
GCASM architecture.

To address this, we propose the GCASM, which ingeniously integrates the strengths of graph convolution in local feature extraction with the advantages of attention mechanisms in global feature extraction. Through this design, GCASM can simultaneously capture both local details and global structural information in plant point clouds, providing richer feature representations for subsequent segmentation tasks. The architecture of GCASM is illustrated in **Figure 4**.

The input to GCASM is a point cloud block of dimension N × C, where N denotes the number of points and C is the number of feature channels specified by the user. First, through Graph Creation Block, the point cloud is modeled as a local map using graph convolution network to extract the geometrically detailed features of plants. Second, through Self-Attention block, the global contextual information is integrated to capture the long-distance semantic associations in the point cloud. Finally, GCASM outputs a feature-updated point cloud block of dimension N×C, in which local details are fully integrated with global contextual information.

#### 2.2.1 Graph creation block

**Figure 5:**
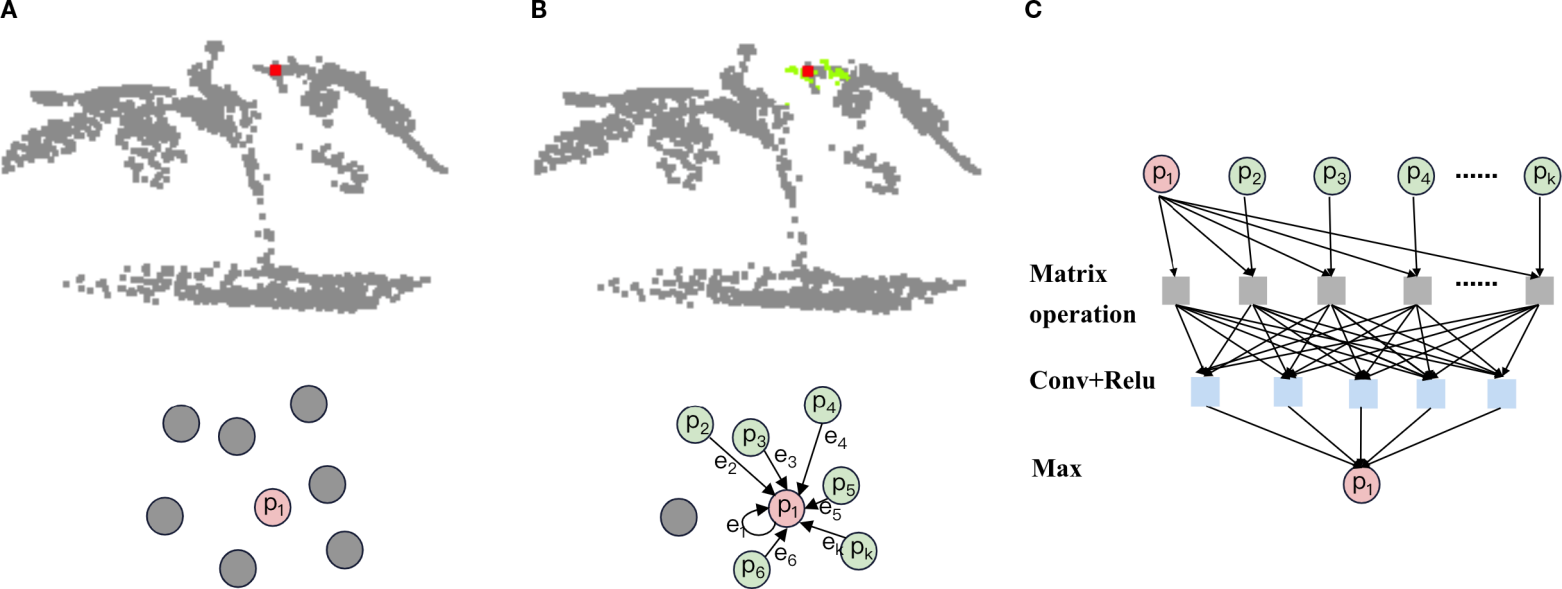
Graph creation block process. **(a)** select the center point:Select a point as the center point for constructing the local graph. **(b)** construct partial drawings: select the k-1 points closest to the center point in terms of Euclidean distance squared to construct a local graph. **(c)** aggregation of local graphs: aggregate the features of neighboring points and edges within the local graph. Replace them with the features of the central point to complete the feature learning integration. The specific content is detailed below.

Graph Convolutional Networks have significant advantages in extracting localized detailed features of plant point clouds. By constructing the graph structure of the point cloud, graph convolution can explicitly model the relationship between each point and its neighbors to accurately capture local geometric structures such as leaf edges, stem curvature, and other detailed features in the plant point cloud (Zhou et al., 2021). At the same time, graph convolution is naturally suited for processing irregular and non-Euclidean-structured point cloud data without the need to convert it to a regular mesh, which can retain more localized detail information (Turgut et al., 2022).The flow of Graph creation block is shown in **Figure 5**.

##### 2.2.1.1 Select the center point

In graph convolution, the purpose of selecting center points is to provide anchor nodes for local graph construction. Acting as the core of a local graph, a center point effectively captures geometric features of local regions in plant point clouds through connectivity with neighboring points. By aggregating local spatial information into the center point, the model’s sensitivity to plant-specific detail features is significantly enhanced.

Common strategies for selecting center points include point-wise selection, random sampling, farthest point sampling, and density-based sampling. Compared to random sampling, point-wise selection avoids uneven coverage of local regions caused by randomness (Li et al., 2023). In contrast to FPS, it eliminates reliance on global point cloud distribution, better adapting to the requirements of local detail extraction. Furthermore, when compared to density-based sampling, point-wise selection avoids the problem of feature deviation caused by density difference.

To effectively address the complex geometric structures of plant point clouds and fully leverage the critical role of graph convolutional networks (GCNs) in local detail extraction, we adopt the point-wise selection strategy. This approach maximizes the retention of plant geometric details by ensuring that every point serves as a center point for local neighborhood feature aggregation. In order to reduce the potential computational inefficiency caused by point by point selection, we applied down sampling to the input point cloud to effectively control the data size. At the same time, we treat a point cloud block as a matrix, which significantly reduces the time consumption of neighborhood computation and ensures the overall efficiency of the algorithm through GPU-accelerated parallel matrix operations.

##### 2.2.1.2 Construct partial drawings

The purpose of constructing local graph structures is to capture local geometric features of plant point clouds by modelling the spatial relationships between points and central points within each local region, thereby enabling effective feature aggregation (Wang et al., 2019). In this way, the model can more accurately describe the local morphological features of plants, and provide high-quality local feature representation for subsequent global feature integration and semantic segmentation tasks.

Using the neighborhood search method, the set of points of a local graph is determined by finding the points in the neighborhood through the determined centroid. The common methods of neighborhood search include Euclidean distance based on distance, cosine similarity based on direction and Pearson correlation coefficient based on statistical relationship (Guo et al., 2003). While Pearson correlation effectively measures feature similarity in high-dimensional spaces, it shows excessive sensitivity to outliers. Cosine similarity focuses on directional alignment but disregards distance information. These limitations make both methods suboptimal for our segmentation requirements. In practical implementation, Euclidean distance demonstrates superior stability and robustness, particularly when processing plant point clouds with natural variations.

In order to further improve the calculation efficiency and reduce the cost of square root operation, we finally choose the square Euclidean distance (Danielsson, 1980) as the index of neighborhood search, as shown below.


$$d^{2}(\vec{x},\vec{y})=\sum_{i=1}^{n}{\left(x_{i}-y_{i}\right)}^{2}$$

Where, the difference between two n-dimensional vectors $\vec{x}=(x_{1},x_{2},\ldots ,x_{n})$ and $\vec{y}=(y_{1},y_{2},\ldots ,y_{n})$ in each dimension, and then sum the squares.

After calculating the square Euclidean distance from the center point to all points in the input point cloud, select K points with smaller index, that is, closer distance to construct the local map. We can represent the local graph as G=(V,E) , where $V=\{p_{i}|i=1,2,\ldots ,k\}$ represents the set of points in the graph, $p_{i}\in {\mathbb{R}}^{1\times C}$ represents the feature information of each point, K represents the number of points, and C represents the feature dimension. Assuming $P_{1}$ is the center point, the edge set $E=\{e_{i}=p_{i}-p_{1}|i=1,2,\ldots ,k\}$ can be obtained by calculating the vector difference between the points in the neighborhood and the center point. - represents the difference of the corresponding dimension element, and $e_{i}\in {\mathbb{R}}^{1\times C}$ represents the edge feature.

##### 2.2.1.3 Aggregation of local graphs

After the local graph is constructed, we need to extract and integrate the information of points and edges in the local graph, so that the spatial geometric information contained in the local graph can be aggregated to the center point to realize the feature representation of the local structure. Since the center point functions as a carrier for aggregating local map information, its intrinsic features should dominate the representation (Zhou et al., 2017). By connecting the center point feature $p_{1}$ and the K edge feature $e_{i}$ on the feature dimension, we generate the spliced feature matrix $F_{\;concate}$ :


$$F_{\;concate}=(p_{1}∥e_{i})\in {\mathbb{R}}^{K\times 2C}$$

Among them, | represents the concatenation operation of feature dimensions, and the dimension of $F_{\;concate}$ is K × 2C.

Following feature stitching, we apply convolutional layers and ReLU activation. This extracts deeper patterns from the combined features, enhancing key feature representation. Since the process alters feature dimensions, we employ max-pooling to aggregate features. This dimensionality restoration maintains compatibility with subsequent attention mechanisms while preserving essential characteristics. The final output is the updated center point feature representation $F_{graph}$ :


$$F_{graph}=max(relu(conv(F_{\;concate})))$$

Where, max represents the maximum pooling, relu represents the ReLU activation function, and conv represents the convolutional layer.

**Figure 6:**
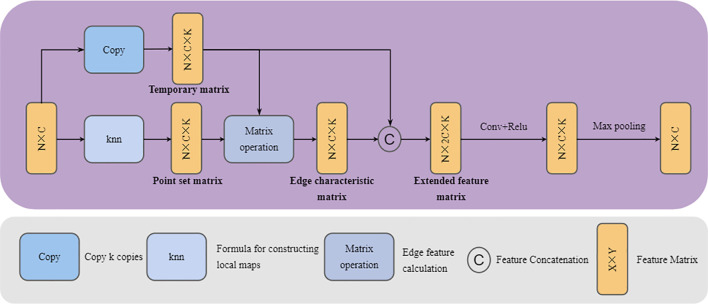
Graph creation block architecture. Copy, Copy the matrix k times and increase the dimension; Knn, Use a k-nearest neighbor algorithm based on square Euclidean distance to construct a local graph. Matrix operation, Use matrix subtraction to construct edge features. The specific content is detailed below.

To sum up, the structure of the Graph creation block can be shown in **Figure 6**. The process can be described in turn as follows: (1) Copy k copies of the original point cloud in the third dimension and store them in a temporary matrix (This matrix stores k copies of the center point features to facilitate subsequent edge feature calculations). (2) For input point cloud data, a point-to-point K-nearest neighbor algorithm based on the squared Euclidean distance is used to construct a point set for the local map. The local map with k points is stored in an N×C×K point set matrix (the center point and its k-1 neighboring points are stored in the third dimension). All point information in the local map is stored in a high-dimensional point set matrix. (3) Subtract the vector between the point set matrix and the temporary matrix on the third dimension to get the edge characteristic matrix. (4) In the third dimension, the temporary matrix is concatenated with the edge feature matrix to form an N×C×2K extended feature matrix. (5) The features on the extended feature matrix are learned and integrated through convolutional layers and ReLU activation functions, and aggregated using a maximum pooling operation to bring them back to their original dimensions.

#### 2.2.2 Self-Attention block

Following feature extraction by the Graph creation block, local detail features of the plant point cloud have been thoroughly explored, yet global contextual information still requires further integration. To address this, we employ a Self-Attention block to perform global modelling of the point cloud features.

**Figure 7:**
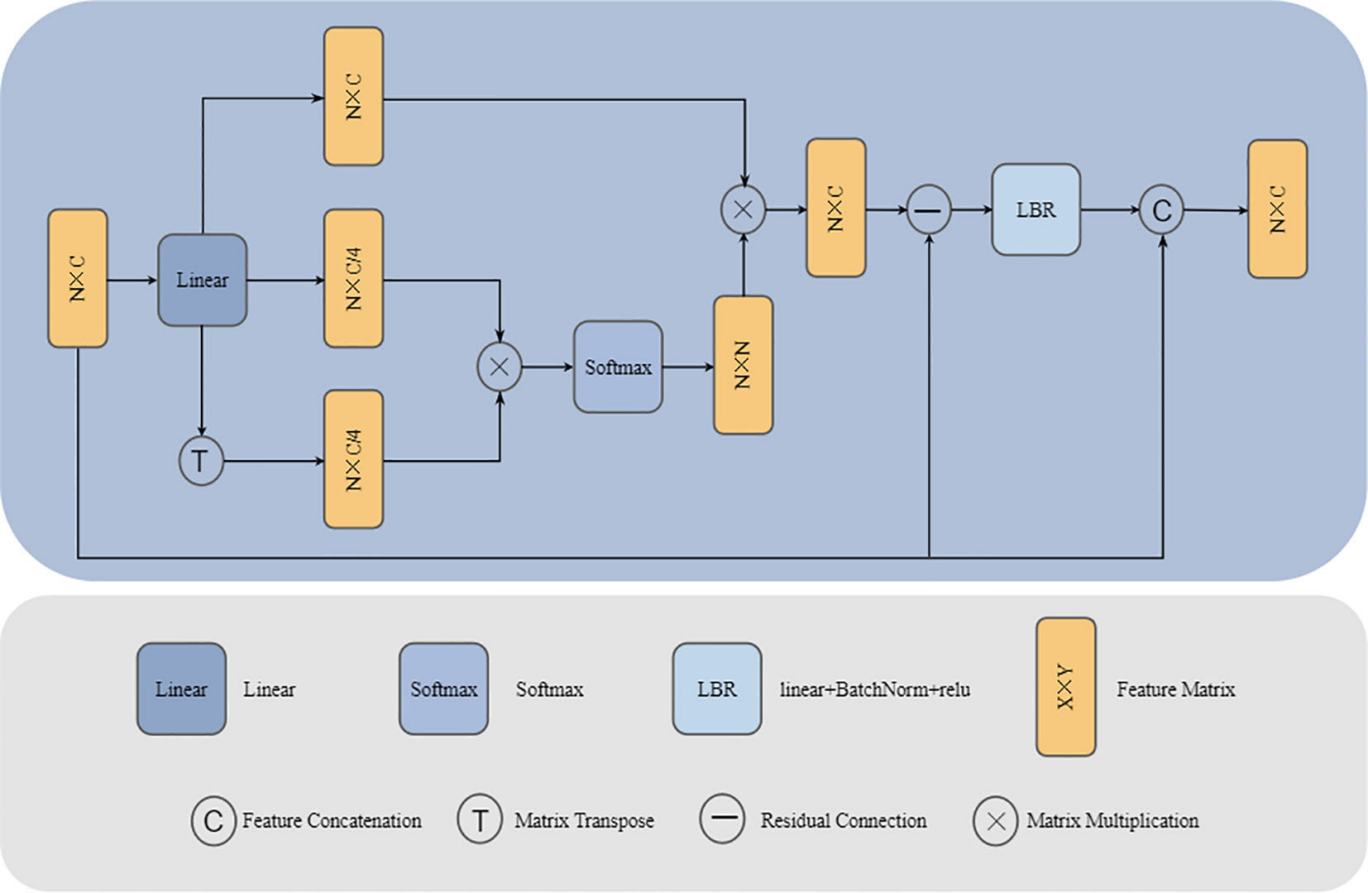
Self-Attention block architecture. Linear, Calculate QKV through linear layers; Softmax, Apply the softmax activation function; LBR, Indicates sequential execution of linear, BatchNorm, and ReLU.

The Self-Attention block effectively captures long-range dependencies within the point cloud by calculating global correlations between points, enabling contextual awareness of the plant’s overall structure (Vaswani et al., 2017). This global feature integration complements the local feature extraction achieved through graph convolution, jointly establishing a multi-level feature representation system that spans from local to global. Consequently, the combined approach provides a comprehensive characterization of the plant’s geometric morphology and structural properties. The architecture of the Self-Attention block is illustrated in **Figure 7**.

First, calculate the query matrix (Q), key matrix (K) and value matrix (V) as follows:


$$\left(Q,K,V)=F_{in}\cdot (W_{q},W_{k},W_{v}\right)$$

$$W_{q}\in {\mathbb{R}}^{N\times C/4},\;W_{k}\in {\mathbb{R}}^{N\times C/4},\;W_{v}\in {\mathbb{R}}^{N\times C}$$

Where, ${\text{F}}_{\text{in}}$ is the input of Self attention block, $W_{q},W_{k},W_{v}$ is the parameter matrix that can be learned.

It is a common operation to scale the dimension of $W_{q},W_{k}$ in the trasnformer model (Wang et al., 2024). Since the dimension of $W_{q},W_{k}$ does not affect the result of calculating Q,K,T , the amount of computation can be greatly reduced by appropriately scaling the dimension of $W_{q},W_{k}$ , and the scaling also makes the distribution of attention more stable. To make the model more lightweight, we scale the dimension of $W_{q},W_{k}$ by a factor of 4 while sharing their parameters.

Then, the similarity between points is calculated through the query matrix (Q) and key matrix (K) to get the attention score:


$$AttentionScore=Q\cdot K^{T}$$

And use Softmax function to normalize it into attention weight:


AttentionWeight=softmax(AttentionScore)

Multiply the attention weight and the value matrix (V), weighted aggregate global features, and generate attention output features:


$$F_{sa}=Attentionweight\cdot V$$

Laplace transform can effectively improve the discrimination ability of feature representation by enhancing the correlation between plant point cloud features (Daxberger et al., 2021).We quantify the differences between different points in the point cloud by constructing an implicit Laplacian matrix ( L=D−E , where D is the diagonal matrix and E is the adjacency matrix). The Laplacian enhancement defined by the diagonal matrix (D) and the adjacency matrix (E) belongs to the category of combinatorial Laplacian enhancements. This is the most basic and widely used form of Laplacian (Guo et al., 2021). For this purpose, we introduce an approximate calculation using the Laplace transform:


$$\begin{array}{c} F_{in}-F_{sa}=F_{in}-Attentionweight\cdot V\\ \;=\;F_{in}-Attentionweight\cdot F_{in}W_{v}\\ \;\approx F_{in}-Attentionweight\cdot F_{in}\\ =(1-Attentionweight)F_{in}\\ \approx (D-E)F_{in} \end{array}$$

we apply it to the output of Self attention block, which reflects the feature enhancement similar to Laplace transform and further improves the representation ability of features. Therefore, the final attention output feature $F_{attention}$ can be expressed as:


$$F_{attention}=LBR(F_{in}-F_{sa})+F_{in}$$

Among them, LBR represents linear layer, BatchNorm normalization layer and ReLU activation function respectively.

So far, through the feature extraction of Graph creation block and Self attention block, our GCASM as a whole can be expressed as:


$$F_{1}=max(relu(conv(x_{1},(x_{i}-x_{1}))))$$

$$F_{2}=softmax((W_{q}\cdot F_{1})\cdot {\left(W_{k}\cdot F_{1}\right)}^{T})\cdot (W_{v}\cdot F_{1})$$

$$F_{out}=LBR(F_{1}-F_{2})+F_{1}$$

Where, ${\text{F}}_{1}$ represents the output features of Graph Creation Block, which fully captures the local details of the plant point cloud, such as leaf edges, stem curvature, and other geometric structures; ${\text{F}}_{2}$ and ${\text{F}}_{\text{out}}$ represent the output features of Self attention block.

## 3 Materials

### 3.1 Plant point cloud data

This experiment utilized two public datasets: Pheno4D (Schunck et al., 2021) and Planest3D (Mertoğlu et al., 2024). The Pheno4D dataset comprises 224 high-resolution 3D point clouds captured daily over a three-week period (starting from the emergence) for seven tomato and maize plants using a high-precision 3D laser scanning system. The annotations include three categories: leaves, stems, and soil. The Planest3D dataset contains point clouds of 10 pepper plants, 14 ribes plants, and 10 rose plants, with annotations limited to two categories: leaves and stems.

**Figure 8:**
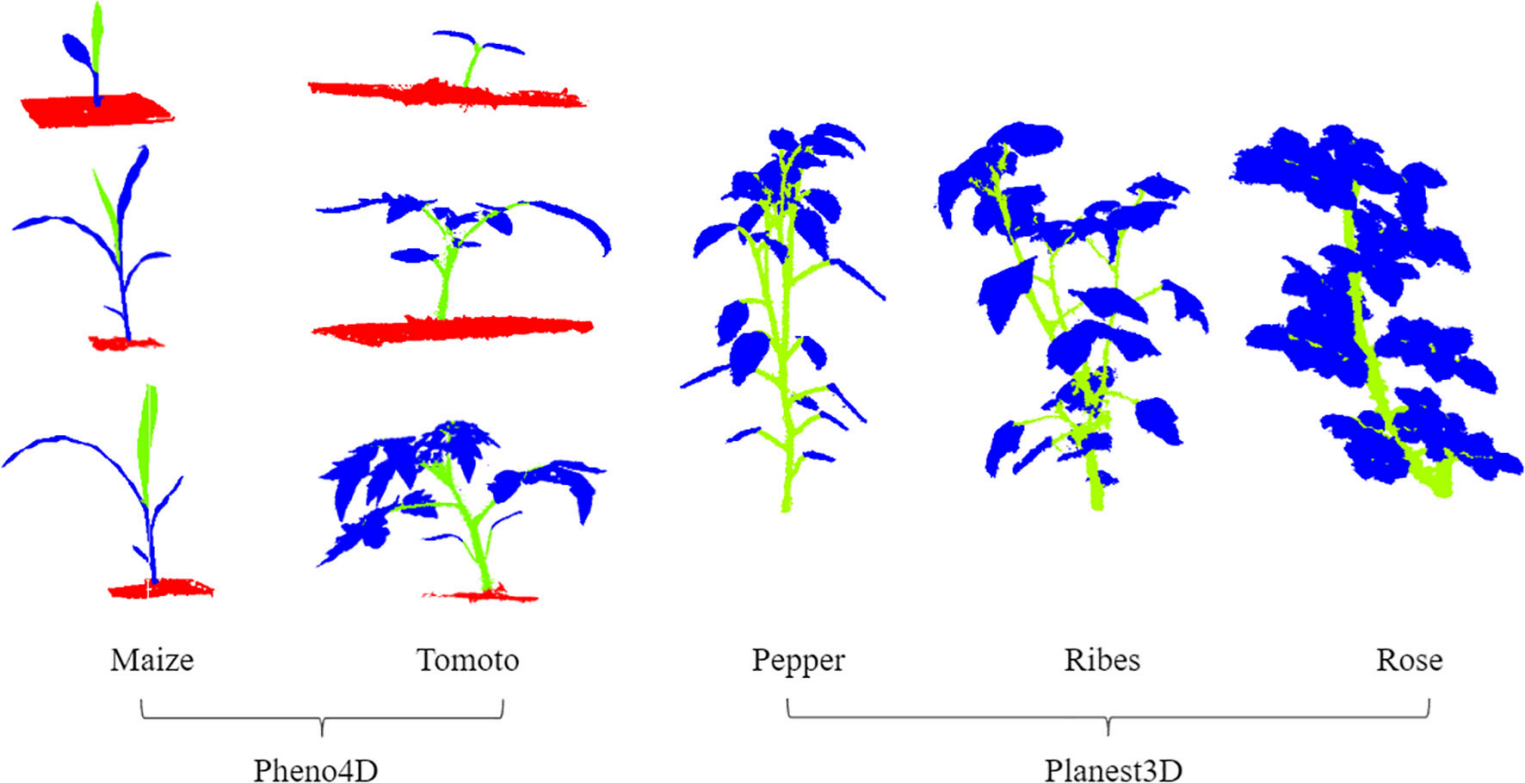
Data presentation of Tomoto, Maize’s three stages, Pepper, Ribes, and Rose’s data presentation(green = stems, blue = leaves, red= soil).

These two datasets are of high quality and contain little noise or irrelevant elements. To facilitate the segmentation task, we manually annotated some of the unlabeled data using the CloudCompare tool. At the same time, we standardized the category representations (stems are represented as 0, leaves as 1, and soil as 3). **Figure 8** illustrates the annotated point cloud samples used in the experiments. The datasets were split into training and test sets in an 8:2 ratio.

Due to the typical presence of millions of points per plant point cloud file, it is impractical to involve all points in computation. Therefore, like many scholars, we chose 2,048 points to represent the characteristics of the entire point cloud. The random sampling method of RandLA-Net has been proven to be efficient and robust, and can significantly improve the performance of large-scale point cloud processing (Hu et al., 2020). Given these advantages, we adopt random sampling for data preprocessing before feeding the data into the segmentation network. Since the plant point cloud data is of high quality and contains very little noise, we did not consider noise processing.

### 3.2 Evaluation metrics

Accuracy (acc) and Mean Intersection over Union (mIoU) are common metrics for evaluating the precision of 3D point cloud semantic segmentation. In 3D point cloud semantic segmentation, the IoU is typically computed for each class, and the mean IoU (mIoU) is derived by averaging these values across all classes.

In a point cloud where each point serves as a training sample, assume the point cloud has n classes labeled as $C_{1}\sum C_{2}\ldots C_{n}$ . For a specific class $C_{i}$ , its Intersection over Union (IoU), denoted as ${\text{IoU}}_{\text{i}}$ , is calculated as:


$$IoU_{i}=\frac{TP_{i}}{TP_{i}+FP_{i}+FN_{i}}$$

Among them, $TP_{i}$ is the number of correctly classified points in category $C_{i}$ , $FP_{i}$ is the number of points in category $C_{i}$ incorrectly classified as positive, $FN_{i}$ is the number of points in category $C_{i}$ that are incorrectly classified as negative. The Mean Intersection over Union (mIoU) is then calculated as:


$$mIoU=\frac{1}{n}\sum_{i=1}^{n}IoU_{i}=\frac{1}{n}\sum_{i=1}^{n}\frac{TP_{i}}{TP_{i}+FP_{i}+FN_{i}}$$

The Accuracy (acc) of each category $C_{i}$ is recorded as $Acc_{i}$ , then the accuracy of n categories can be expressed as:


$$Acc=\frac{1}{n}\sum_{i=1}^{n}Acc_{i}=\frac{1}{n}\sum_{i=1}^{n}\frac{TP_{i}+TN_{i}}{TP_{i}+FP_{i}+FP_{i}+FN_{i}}$$

$TN_{i}$ is the number of points correctly classified as negative category in category $C_{i}$ .

## 4 Results

All experiments were conducted on a host machine with the following configuration: a 12th Gen Intel^®^ Core™ i5-9300H CPU (8 cores) operating at 2.4GHz base frequency, paired with an NVIDIA GeForce RTX 1650 GPU (4GB VRAM). The system utilized CUDA 11.8 for acceleration and ran on Windows 10 OS. The deep learning framework employed PyTorch 2.2.1.

For all model experiments, consistent hyperparameters were maintained: batch size set to 8 (Small batches can lead to better generalization capabilities.), initial learning rate of 0.1 (0.1 is a typical initial value.), and training duration of 200 epochs. The learning rate was multiplied by 0.5 every 20 epochs (Learning rate decay strategies are crucial for model convergence and fine-tuning in the later stages.). Training utilized the Stochastic Gradient Descent (SGD) optimizer with momentum 0.9 and weight decay 0.0001 (Very commonly used values, which usually work well and are robust.). The final reported results correspond to the epoch that achieved the highest mean Intersection over Union (mIoU) performance on the test set.

### 4.1 Comparative experiment

**Figure 9:**
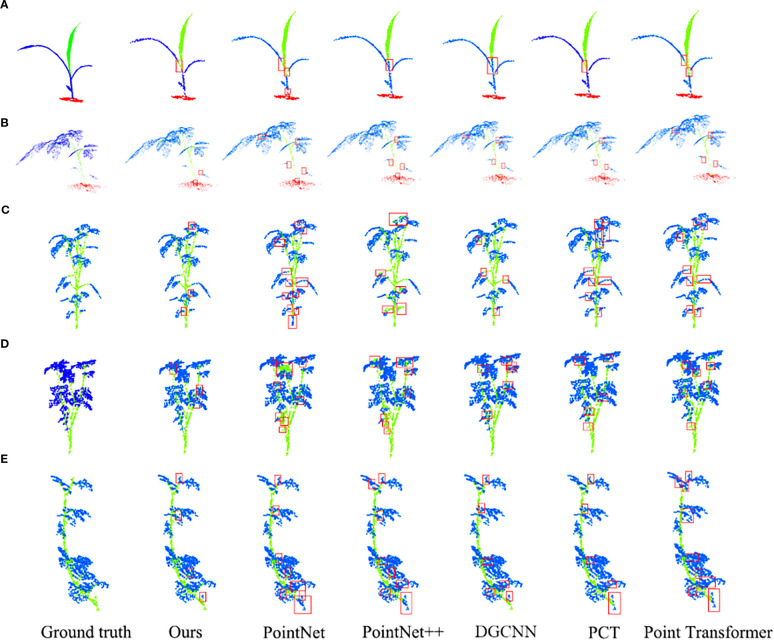
Segmentation visualization. **(a)** Maize. **(b)** Tomato. **(c)** Pepper. **(d)** Ribes. **(e)** Rose. The red box represent the error identification part.

We selected several representative methods (PointNet (Qi et al., 2017b), PointNet++ (Qi et al., 2017b), DGCNN (Wang et al., 2019), Point Transformer (Zhao et al., 2021), PCT (Guo et al., 2021)) and conducted comparative experiments under identical conditions with our proposed method. The evaluation focused on two dimensions: segmentation accuracy and computational efficiency. All models were tested on multiple plant point cloud datasets (Pheno4D and Planest3D) to measure Accuracy (acc) and Mean Intersection over Union (mIoU). Quantitative results are summarized in the **Table 1**, and qualitative visualizations are provided in **Figure 9**.

**Table 1:** Comparison of the experimental results.

Model	acc	mIoU	Pheno4D				Planest3D					
			Tomato		Maize		Pepper		Ribes		Rose	
			acc	mIoU	acc	mIoU	acc	mIoU	acc	mIoU	acc	mIoU
Pointnet	88.37	57.88	96.93	77.02	95.02	60.00	86.53	50.20	80.92	51.11	82.47	51.09
Pointnet++	94.27	72.57	97.73	83.62	**97.53**	**76.45**	92.10	68.22	92.02	67.18	91.96	67.39
DGCNN	94.44	87.50	97.59	84.03	95.75	66.41	95.41	96.90	92.93	95.74	90.53	94.41
Point Transformer	92.30	87.76	93.85	85.99	93.09	68.24	91.33	94.93	90.23	94.65	91.99	94.99
PCT	91.80	85.19	95.76	82.56	93.55	62.42	92.33	94.95	91.37	94.5	85.99	91.54
Ours	**95.46**	**90.41**	**97.89**	**88.38**	97.32	74.60	**95.60**	**97.08**	**93.70**	**96.54**	**92.77**	**95.45**

The best results for each metric are highlighted in bold.

On the whole, our model is superior to other comparative models in acc and mIoU. Our model achieves 1 percentage point higher accuracy than DGCNN and 2.7 percentage points higher mIoU than Point Transformer. This shows that our model has stronger ability in global and local feature extraction, and can segment complex plant point cloud data more accurately. In addition, our model also performs well in operational efficiency. Only slightly lower than DGCNN, significantly better than attention based methods (Point Transformer, PCT), achieving a good balance between accuracy and efficiency.

Although our model performs well on most plant types, its performance on Maize is slightly lower than that of Pointnet++. The specific reason is that Maize’s stems and leaves are not clearly separated, and the plant does not show clearly identifiable stems, which makes it difficult for the model to accurately distinguish stems and leaves during segmentation. This phenomenon suggests that the task of segmenting maize is highly challenging.

From the comparison of models, the segmentation effect of PointNet series is poor under the condition of high time consumption, especially the mIoU (PointNet:57.88%, PointNet ++:72.57%) is significantly lower than our model. PointNet, as an early point cloud processing method, is weak in local feature extraction, and it is difficult to capture the leaf edge, stem curvature and other details in the plant point cloud. While PointNet++ advances beyond PointNet by employing hierarchical feature extraction to better describe local plant geometries, its global feature representation capacity remains inadequate. For instance, on the maize dataset, PointNet++ outperforms our model, likely due to the relatively simple plant structure and primarily relies on local geometric features. However, it is common in other plant types, indicating that these plant types need stronger ability to understand the global context.

While DGCNN demonstrates the lowest computational time, its accuracy and mIoU trail our model by 1.02 and 2.91 percentage points respectively. DGCNN extracts local features through dynamic graph convolution, which is relatively stable and can better capture local geometric information in plant point clouds, but it is still inferior to our model in capturing global features.

Attention mechanism-based methods (Point Transformer and PCT) are not only more time-consuming but also less accurate (in terms of acc and mIoU) than our model. Point Transformer can effectively extract local global features by dynamically aggregating and coding point clouds through multi head self attention mechanism, but its complex structure makes it obtain more redundant information when dealing with plant point clouds. PCT also employs the self-attention mechanism, but it performs weakly in the extraction of local features and is difficult to accurately characterize the detailed structures in the plant point cloud. So, it does not perform as well as our model in the task of segmenting complex plant point clouds.

In addition, we recorded the inference time required for each model to process a plant point cloud block (2,048 points, ~98 KB data) and the number of parameters in the model. These metrics were used to measure the efficiency of the model. The relevant data is shown in **Table 2**.

**Table 2:** Comparison of efficiency parameters.

Model	Times(s)	Params
Pointnet	0.709	8342395(8M)
Pointnet++	0.732	1740958(2M)
DGCNN	**0.670**	**1454217(1M)**
Point Transformer	2.741	19397362(19M)
PCT	1.185	2433794(2M)
**Ours**	0.691	1512585(2M)

The best results for each metric are highlighted in bold.

In inference efficiency tests on a single plant point cloud (2,048 points), our model achieved an efficient balance between speed and lightweight design with an inference time of 0.691 s and a complexity of 1.5 million parameters (approximately 2 million). Its inference speed is second only to the fastest DGCNN (0.670 s), while its parameter count is significantly lower than that of PointNet (8 million) and Point Transformer (19 million), and comparable to lightweight models such as PointNet++ and PCT.

In general, our GCASSN can simultaneously capture local details (such as leaf shape, stem curvature) and global structure (such as overall shape, branch topology) in the plant point cloud. It showing high accuracy and robustness in the plant point cloud segmentation task, and saving time and parameter consumption.

In the visual analysis, there are many wrong segmentation at the junction in other comparison models, such as PointNet segmentation for Pepper (**Figure 9c**), DGCNN segmentation for tomato (**Figure 9b**), PCT segmentation for ribes (**Figure 9d**), etc. In addition, there are obvious error recognition in dense areas, such as the segmentation of rose (**Figure 9e**) dense areas by PointNet, and the segmentation of ribes (**Figure 9d**) dense areas by Point Transformer.

Our model mainly makes slight segmentation errors at the junction of some stems and leaves, such as the junction of stems and leaves of tomato (**Figure 9b**), pepper (**Figure 9c**) and rose (**Figure 9e**). These errors are usually small in scope, and the reason for errors at this junction is closely related to plant characteristics. At the junction of stem and leaf, the geometric structure and feature distribution are often very similar. For example, the stem gradually transits to the petiole, and the curvature and surface texture change are relatively smooth, which makes it difficult to distinguish models clearly. At the same time, there are some recognition errors in the dense areas of leaves, such as the dense areas of maize (**Figure 9a**), ribes (**Figure 9d**), rose (**Figure 9e**). This is because the density and distribution of point cloud data at the junction may be uneven, which further increases the difficulty of model learning.

### 4.2 Cross-species generalization validation

In order to evaluate the generalization ability of the network, this study designed a cross species validation experiment. The experiment selected Pepper in Plant3D dataset and Tomato in Phone4D dataset as the experimental objects. The two species have certain similarities in plant morphology and organ structure, such as obvious stem structure, branching mode and leaf distribution characteristics, but there are also differences at the same time. For example, tomato leaves are usually larger and more complex, while pepper stems are thinner and more branched. This combination of similarity and difference provides an ideal experimental condition for evaluating the generalization ability of the model among different plant species. In the data preprocessing phase, we standardized two data sets: (1) remove the soil background information contained in the tomato data set; (2) Unify the labeling standards of stems and leaves in the two datasets.

**Figure 10:**
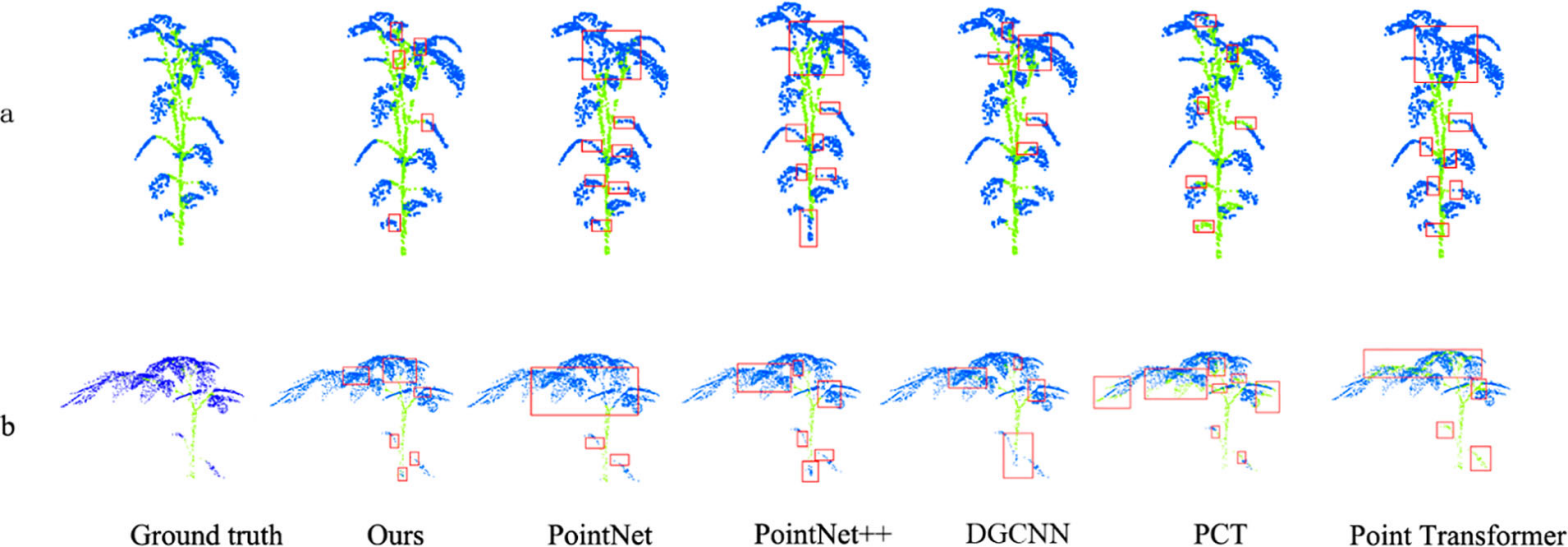
Cross experiment visualization, with the red box represent the error identification part.

We implement two strategies: (A) training the network on the pepper dataset and testing on the tomato dataset. (B) The network was trained on the tomato dataset and tested on the pepper dataset. The relevant experimental results are shown in the **Table 3** and visualized in the **Figure 10**.

**Table 3:** Cross-Species Generalization Validation Experiment.

Model	A		B	
	acc	mIoU	acc	mIoU
Pointnet	91.28	60.39	83.98	68.21
Pointnet++	91.69	55.2	93.28	84.73
DGCNN	88.87	58.09	92.84	84.41
Point Transformer	89.73	57.01	91.45	84.1
PCT	87.32	52.92	90.16	78.9
**Ours**	**91.45**	**60.8**	**95.19**	**88.66**

The best results for each metric are highlighted in bold.

From the overall results, the segmentation of B (Tomato→Pepper) is slightly better than that of A (Pepper→Tomato). This may be due to the relatively clear leaf structure of Pepper, whereas the leaves of Tomato are denser and more morphologically complex.

The comparison experiment results show that our model is superior to other comparison models in Acc and mIoU regardless of the setting of A (Pepper → Tomato) or B (Tomato → Pepper). This advantage is due to the fact that our model can capture local details and global structural information of pepper and tomato at the same time, so as to better adapt to the morphological differences of different plant species. In contrast, other models cannot achieve an effective balance between local and global feature extraction.

In the visualization results, the baseline model exhibited large-scale errors in dense leaf areas and stem-leaf junctions. For instance, it failed to correctly segment the top dense regions of both pepper and tomato plants (**Figure 10a**). In contrast, while our model still has minor errors in these challenging regions (**Figures 10a, d**), it significantly outperforms the baseline, demonstrating robust cross-species generalization for stem-leaf segmentation. This ability enables the model to recognize the general characteristics of stems and leaves of different plants, thus maintaining high segmentation accuracy in complex scenes.

Our method achieves state-of-the-art performance in two-way cross-validation, particularly in mIoU, while producing clearer and more accurate segmentation in visual results. This demonstrates the effectiveness of our design, which combines graph convolution networks with attention mechanisms for cross-species point cloud segmentation. Experiments confirm that our approach not only captures fine local details but also handles interspecies morphological variations through global feature modelling.

### 4.3 Generalization evaluation on ShapeNet dataset

In order to further explore the generalization ability and robustness of the network built in this paper, we selected the ShapeNet dataset (Yi et al., 2016) to carry out semantic segmentation experiments. ShapeNet data set is a large-scale 3D shape data set with 16880 3D point clouds, covering 16 categories and 50 annotation parts. Each point cloud contains 2 to 6 parts. ShapeNet data is divided into 14006 model training sets and 2874 model test sets.

The semantic segmentation results of each object in the ShapeNet dataset are shown in **Table 4**, showing each category mIoU, mcIoU and average mIoU. mcIoU is the average mIoU of all shape categories. mcIoU is obtained by calculating the average mIoU of all test cases. The results of the comparison network are from the original paper.

**Table 4:** Results of ShapeNet dataset.

Network	mcIoU	mIoU	aero	bag	cap	car	chair	Ear phone	guitar	knife	lamp	laptop	motor	mug	pistol	Rocket	Skate board	table
Pointnet	80.4	83.7	83.4	78.7	82.5	74.9	89.6	73	91.5	85.9	80.8	95.3	65.2	93	81.2	57.9	72.8	80.6
Pointnet++	81.9	85.1	82.4	79	87.7	77.3	90.8	71.8	91	85.9	83.7	95.3	**71.6**	94.1	81.3	58.7	76.4	82.6
DGCNN	82.3	85.2	84	83.4	86.7	77.8	90.6	**74.7**	91.2	87.5	82.8	95.7	66.3	94.9	81.1	63.5	74.5	82.6
Point Transformer0	**83.7**	**86.6**	**-**	–	**-**	**-**	**-**	–	**-**	–	**-**	**-**	–	**-**	**-**	–	**-**	**-**
PCT	83.1	86.4	**85**	82.4	**89**	**81.2**	**91.9**	71.5	**91.3**	88.1	**86.3**	**95.8**	64.6	**95.8**	**83.6**	62.2	**77.6**	**83.7**
Ours	82.9	85.7	84.1	**84.8**	88.5	77.7	91	73	91.2	**88.6**	85.5	94.9	68	94.1	82.2	**64**	75	83

The best results for each metric are highlighted in bold.

Without targeted optimization, our GCASSN shows strong comprehensive performance. In terms of overall indicators, although slightly lower than PCT and Point Transformer, it is outstanding in several key categories. For example, it achieves the highest accuracy on bag, knife and rocket, which is better than the comparison model, indicating that the model may have a strong ability to capture local details (such as handles and blades) and regular geometry (such as rocket structure). Notably, the model’s performance approaches the optimal performance of PCT on high-complexity categories like chairs and guitars. This demonstrates that its design, combining graph convolution with an attention mechanism, effectively balances local and global features. Compared with the benchmark model, the model ranked first in 3 of the 16 categories, and second in 8 categories. Its overall performance was close to PCT and Point Transformer, indicating that the model’s infrastructure and algorithm had certain effectiveness and adaptability.

## 5 Discussion

### 5.1 Ablation study on key modules

The ablation experiment in this part mainly focuses on exploring the importance of different modules in our method. Observe the changes in model performance by removing Trans-net, Graph creation block, and Self-Attention block. We deeply analyze the contribution of each module to the overall segmentation effect. The specific results are shown in the following **Table 5**:

**Table 5:** Ablation study on key modules.

Operation	acc	mIoU	Pheno4D				Planest3D					
			Tomato		Maize		Pepper		Ribes		Rose	
			acc	mIoU	acc	mIoU	acc	mIoU	acc	mIoU	acc	mIoU
Remove the Trans-net	92.74	87.88	97.77	85.43	97.01	72.13	92.17	95.48	89.11	93.63	87.65	92.73
Remove Graph creation block	87.85	80.69	96.63	75.13	93.35	62.46	86.55	91.95	83.53	87.50	79.17	86.43
Remove Self-Attention block	93.16	88.43	**98.01**	86.82	95.05	69.37	94.90	96.60	91.02	95.00	86.83	94.37
complete	**95.46**	**90.41**	97.89	**88.38**	**97.32**	**74.60**	**95.61**	**97.08**	**93.70**	**96.54**	**92.77**	**95.45**

The best results for each metric are highlighted in bold.

Removing the Trans-net significantly reduces model performance, with acc decreasing by 2.72% and mIoU by 2.53%. These results show that the Trans-net shows strong adaptability to point clouds captured from different angles and attitudes. By improving Spatial Transformation invariance, the model achieves greater robustness and generalization. In practical applications, the pose of point clouds may vary due to different acquisition equipment or plant growth status. The pose adjustment module provides more stable input for subsequent feature extraction by standardizing the spatial distribution of point clouds.

After removing the graph construction module, the model exhibits the most significant performance degradation, with acc dropping by 7.61% and mIoU by 9.72%. This demonstrates the critical importance of the graph creation block. Graph convolution effectively extracts local features while capturing spatial relationships and topological structures. Without graph creation block, the model may not be able to accurately capture the geometric structure of the point cloud, thus affecting the segmentation results.

Self-Attention block can help model extract global features and improve the expression ability of features. After removing this module, the acc decreasing by 2.3%, and mIoU by 1.98%. The small impact suggests that it plays a supporting role in the overall segmentation process. But its approximately two percentage point improvement also represents its successful extraction of global contextual information, improving the model’s feature perception at the global level.

At the same time, we recorded data on the number of parameters and time consumption after removing each module. Through this data, we can better measure the consumption of each module. The relevant data is shown in **Table 6**.

**Table 6:** Comparison of efficiency parameters.

Model	Times(s)	Params
Remove the Trans-net	0.632	673920 (1M)
Remove Graph creation block	0.609	1495817 (1M)
Remove Self-Attention block	**0.573**	**1484169 **(1M)
complete	0.691	1512585 (2M)

The best results for each metric are highlighted in bold.

Removing the Trans-net module (0.632s, 670,000 parameters) reduces the parameters by 55% and the time by 9%, indicating that this module is the main source of parameters but has little impact on computational efficiency; Removing the Self-Attention module (0.573s, 1.48 million parameters) achieved the greatest time reduction (17%), proving that it is the core computational bottleneck; removing the Graph module (0.609s, 1.49 million parameters) resulted in a relatively balanced reduction in parameter and time. The experiments show that Self-Attention is the key constraint on computational efficiency, Trans-net drives the increase in parameter count, and the Graph module has a relatively balanced impact.

The ablation experiment results show that the Trans-net, graph creation block and Self-Attention block are all important for model performance. Among them, the Trans-net enhances the robustness of the model by normalizing the spatial distribution of point clouds. The graph creation block is very important for local feature extraction and geometric structure modelling. The Self-Attention block further improves the global awareness of the model by integrating global features. This three factors work together to make the model achieve excellent performance in 3D point cloud segmentation of complex plants.

### 5.2 Layerwise ablation study for network depth

In deep learning, the level of feature extraction module has a significant impact. Less layers may not be able to fully extract complex features, resulting in poor model performance. However, if the number of layers is too many, it may cause problems such as over fitting and gradient disappearance. In order to determine the optimal number of layers of the GCASM module, we have taken different layers for experiments. The specific results are shown in the **Table 7**.

**Table 7:** Layerwise ablation study for network depth.

Number of layers	acc	mIoU	Pheno4D				Planest3D					
			Tomato		Maize		Pepper		Ribes		Rose	
			acc	mIoU	acc	mIoU	acc	mIoU	acc	mIoU	acc	mIoU
1	92.25	85.77	97.55	85.02	95.99	61.91	91.70	94.77	86.38	93.04	89.62	94.09
2	93.96	88.84	97.54	86.56	97.17	72.26	93.90	96.04	92.84	96.14	88.35	93.18
3	**95.46**	**90.41**	**97.89**	**88.38**	**97.32**	**74.60**	**95.61**	**97.08**	**93.70**	**96.54**	**92.77**	**95.45**
4	92.47	82.69	94.83	63.89	91.59	63.87	94.65	96.46	93.60	96.52	87.67	92.73

The best results for each metric are highlighted in bold.

The experimental results demonstrate that as the number of GCASM module layers increases from 1 to 3, the point cloud segmentation performance gradually improves, with accuracy and mIoU increasing by 1.71% and 1.5% for the first layer, and 3.07% and 1.57% for subsequent layers, respectively. This improvement suggests that additional GCASM layers enable the model to learn richer feature representations within an optimal range, thereby enhancing its ability to distinguish among different plant parts. When the layer count reaches 4, the segmentation performance deteriorates significantly, with accuracy and mIoU dropping by 2.99% and 7.72%, respectively. This performance decline occurs because excessive model complexity leads to information overload, making it difficult for the model to extract meaningful features.

At the same time, we recorded the time and parameter consumption for different numbers of layers. The relevant results are shown in **Table 8**.

**Table 8:** Comparison of efficiency parameters.

Number of layers	Times(s)	Params
1	0.556	1361033 (1M)
2	0.649	1442953 (1M)
3	0.691	1524873 (2M)
4	0.73	1624457 (2M)

Experiments have shown that the size of model parameters increases steadily and linearly with the number of layers, with approximately 82000 to 100000 parameters introduced for each new layer. The total number of parameters in layer 4 (1.62M) only expands by 19.1% compared to layer 1 (1.36M); The increase in inference time exhibits nonlinear characteristics: the time consumption significantly increases by 16.7% (+0.093s) from layer 1 to layer 2, but the marginal time cost gradually decreases for each additional layer, indicating that deep models can more efficiently utilize computing resources. Overall, moderately increasing the number of layers can improve the model capacity with limited parameter increments, and deep structures have better computational efficiency.

Therefore, the number of layers of GCASM module needs to be optimized within a certain range to balance the complexity of the model and the ability of feature extraction. Comprehensive consideration of segmentation effects and consumption, the 3-tier structure can capture complex geometric features while avoiding information overload, so as to achieve more accurate segmentation.

### 5.3 Comparative spatial transformation modules

In the task of point cloud segmentation, spatial location awareness is the key factor to ensure the performance of the model. The models we compare realize the perception of point cloud pose through different strategies: (1) PointNet uses the dual affine transformation mechanism. The original point clouds are aligned in spatial coordinates through the 3 × 3 affine transformation matrix predicted by T-Net, and then a 64 × 64 feature transformation matrix is constructed to correct the high-dimensional feature space. (2) DGCNN innovatively introduces the dynamic graph convolution structure, and its Spatial Transformation module can adaptively learn the distribution characteristics of transformed point clouds. (3) Other representative networks use implicit spatial alignment strategies. For example, PointNet++ builds multi-scale spatial awareness through hierarchical feature extraction architecture. PCT and Point Transformer embed spatial coordinate information into high-dimensional feature representation through position coding technology. These methods essentially work by establishing a stable mapping relationship between the coordinate system and the feature space.

Trans-net is similar to PointNet’s T-net and DGCNN’s Spatial Transform. After embedding the above two and our Trans-net into our model, the corresponding results are presented in **Table 9**. Our Trans-net achieves superior performance (acc: 95.46%, mIoU: 90.41%).

**Table 9:** Comparative spatial transformation modules.

Operation	acc	mIoU	Pheno4D				Planest3D					
			Tomato		Maize		Pepper		Ribes		Rose	
			acc	mIoU	acc	mIoU	acc	mIoU	acc	mIoU	acc	mIoU
Remove Trans-net	92.74	87.88	97.77	85.43	97.01	72.13	92.17	95.48	89.11	93.63	87.65	92.73
Replace with T-net	92.17	86.84	97.84	84.98	92.16	65.66	93.85	95.95	90.64	94.99	86.38	92.61
Replace with Spatial Transform	94.21	88.15	98.17	85.99	94.95	67.56	94.43	96.43	93.32	95.97	90.19	94.81
Ours	**95.46**	**90.41**	**97.89**	**88.38**	**97.32**	**74.60**	**95.61**	**97.08**	**93.70**	**96.54**	**92.77**	**95.45**

The best results for each metric are highlighted in bold.

The limitation of T-net is that it only realizes attitude adjustment through full connection layer, and lacks the ability to model the local geometric structure of point cloud. This makes it difficult to accurately capture the Spatial Transformation relationship of point clouds when processing complex plant point clouds. Spatial Transform also uses the idea of graph convolution, which can capture the local geometric characteristics of the point cloud. However, it lacks the ability to integrate global context information, resulting in its poor segmentation performance in complex regions.

Since T-net and Spatial Transform are all analogous to a small PointNet or DGCNN. T-net (based on convolution), Spatial Transform (based on graphical convolution) and Trans-net (based on graphical convolution-self-attention) are progressively better fitted to the GCASSN, while the results were positively correlated with the degree of match. This reflects the fact that it is more effective to choose attitude adjustment modules that are compatible with the overall network architecture. On the contrary, if attitude adjustment module or inappropriate attitude adjustment module is used, it may bring negative effects to the model and adversely affect its performance.

### 5.4 Ablation study on graph construction scale

In our GCASM, graph convolution is an important part, and the core of graph convolution is the construction of local graphs. Among them, the parameter k is the number of neighbor points in the local graph, which directly determines the size of the local graph. This size plays a crucial role in the extraction of local information. Starting from obtaining better local features, we set different k values to explore the impact of local map size on the model. The specific results are shown in **Table 10**.

**Table 10:** Ablation study on graph construction scale.

K	acc	mIoU	Pheno4D				Planest3D					
			Tomato		Maize		Pepper		Ribes		Rose	
			acc	mIoU	acc	mIoU	acc	mIoU	acc	mIoU	acc	mIoU
10	93.42	88.47	96.84	84.48	96.25	73.00	94.21	96.16	92.69	96.13	87.09	92.61
20	94.40	88.55	97.65	86.89	96.66	69.18	94.97	96.63	92.74	96.04	89.97	94.01
30	**95.46**	**90.41**	**97.89**	**88.38**	**97.32**	**74.60**	**95.61**	**97.08**	**93.70**	96.54	**92.77**	**95.45**
40	94.79	88.97	97.34	83.76	97.12	73.37	95.09	96.75	93.64	**96.55**	90.77	94.44

The best results for each metric are highlighted in bold.

We set the value of parameter k to 10, 20, 30 and 40 respectively to carry out the experiment. When k=10, the model achieves 93.42% acc and an mIoU of 88.47%, reflecting relatively low performance. This suggests that the smaller local map size may fail to fully capture structural details in plant point clouds, leading to insufficient feature extraction. As k increases to 20, the model’s performance improves, demonstrating that expanding the local map size enhances local feature extraction. The optimal performance occurs at k=30, where the model reaches 95.46% acc and 90.41% mIoU, effectively balancing local and global feature learning. However, when k=40, performance declines (acc: 94.79%, mIoU: 88.97%), indicating that excessive neighboring points introduce redundant information and weaken the model’s focus on discriminative local features.

The fluctuations in the experimental results fully demonstrate the significance and value of choosing the appropriate k value for different plant point cloud segmentation tasks. A suitable local map scale can help the model better capture the local geometric features of the plant point cloud, thus improving the segmentation performance.

### 5.5 Optimal metrics for local graph construction

Another key point of graph convolution is to build the evaluation index of local graph. We conducted experiments for three evaluation metrics (Square Euclidean distance, Pearson correlation coefficient and Cosine similarity). At the same time, the combination of squared Euclidean distance and cosine similarity is able to consider distance and direction at the same time. We also experiment with the two indicators with different weight combinations. We adopt the following strategies: (A) Square Euclidean distance. (B) Pearson correlation coefficient. (C) Cosine similarity. (D) 0.5 * Square Euclidean distance+0.5 * Cosine similarity. (E) 0.7 * Square Euclidean distance+0.3 * Cosine similarity. The specific results are shown in the **Table 11**.

**Table 11:** Optimal metrics for local graph construction.

Operation	acc	mIoU	Pheno4D				Planest3D					
			Tomato		Maize		Pepper		Ribes		Rose	
			acc	mIoU	acc	mIoU	acc	mIoU	acc	mIoU	acc	mIoU
A	**95.46**	**90.41**	**97.89**	**88.38**	**97.32**	**74.60**	**95.61**	**97.08**	**93.70**	**96.54**	**92.77**	**95.45**
B	86.39	71.78	85.82	69.58	96.23	65.53	87.93	80.98	91.03	70.44	70.92	72.36
C	90.71	75.22	88.81	75.97	96.54	65.22	88.51	82.75	92.35	72.46	87.33	79.71
D	90.43	75.57	87.35	75.99	94.65	67.05	89.21	79.11	91.66	73.86	89.30	81.84
E	91.30	77.94	90.16	80.95	94.36	67.76	91.11	83.97	91.75	72.64	89.12	84.40

The best results for each metric are highlighted in bold.

The experimental results clearly show that the square Euclidean distance is clearly superior to other evaluation indicators. This phenomenon strongly indicates that in the process of local map division, the influence of distance factor is more prominent than that of spatial similarity and direction factor. We try to combine the squared Euclidean distance and cosine similarity. Experimental results demonstrate that model performance improves progressively with increasing square Euclidean distance weight. When using equal weights (0.5:0.5), the model achieves 90.43% acc and 75.57% mIoU. Adjusting the weights to 0.7:0.3 yields further improvements, increasing acc to 91.30% and mIoU to 77.94%. This trend is consistent with the results of a single index, which further proves that the square Euclidean distance plays a leading role in local map division. The distance factor can more effectively capture the spatial distribution and local geometric characteristics of point clouds.

In conclusion, the square Euclidean distance has significant advantages in local map division, which can more accurately reflect the spatial relationship of point clouds. In contrast, Pearson correlation coefficient and cosine similarity are difficult to achieve ideal results in the plant point cloud segmentation task due to their dependence on directional information.

### 5.6 Attention mechanism comparison

In this study, we conducted a confirmatory comparative experiment on the general self attention mechanism and the multi head attention mechanism, aiming to explore the effects of different attention mechanisms on plant point cloud data processing. The detailed results are listed in the **Table 12**. The general self-attention comes from the Point Transformer. Multi-head attention is based on general self-attention and is obtained following the generalized process of standard multi-head attention.

**Table 12:** Attention mechanism comparison .

Operation	acc	mIoU	Pheno4D				Planest3D					
			Tomato		Maize		Pepper		Ribes		Rose	
			acc	mIoU	acc	mIoU	acc	mIoU	acc	mIoU	acc	mIoU
General self attention	93.51	86.32	94.88	87.92	94.61	68.43	93.73	92.02	93.18	92.29	91.13	90.96
Multi head attention mechanism(4 head)	91.85	80.16	94.43	86.32	92.35	66.32	91.66	86.63	91.44	79.33	89.35	82.22
Multi head attention mechanism(8 head)	91.79	79.15	92.56	85.59	93.30	65.59	91.09	83.89	92.09	76.69	89.89	84.01
Ours	**95.46**	**90.41**	**97.89**	**88.38**	**97.32**	**74.60**	**95.61**	**97.08**	**93.70**	**96.54**	**92.77**	**95.45**

The best results for each metric are highlighted in bold.

The experimental results show the following two significant characteristics: (1) The acc and mIoU of the self attention mechanism are better than those of the multi head attention mechanism. (2) Our attention design outperforms traditional self-attention by 1.95% (acc) and 4.09% (mIoU).

General self-attention has limited ability to model local features, although it can integrate global information. Multi-head attention destroys the consistency of local features extracted by graph convolution due to the interference of multiple branching structures. Our method enhances geometric features through Laplace transform, which can better capture structural features in plant point clouds.

### 5.7 Robustness to varying point cloud density

To evaluate the model’s robustness to varying point cloud densities, we conducted systematic experiments by randomly removing points from the input data. This approach allows us to quantitatively assess the segmentation performance under different density conditions, with detailed results presented in **Table 13**.

**Table 13:** Robustness to varying point cloud density.

Discard quantity	acc	mIoU	Pheno4D					Planest3D				
			Tomato		Maize		Pepper		Ribes		Rose	
			acc	mIoU	acc	mIoU	acc	mIoU	acc	mIoU	acc	mIoU
Discard 0	**95.46**	**90.41**	97.89	**88.38**	97.32	**74.60**	**95.61**	**97.08**	93.70	**96.54**	**92.77**	**95.45**
Discard 512	94.28	89.46	**97.97**	88.34	**97.33**	73.91	92.02	94.80	**93.87**	96.05	90.19	94.21
Discard 1024	94.03	88.95	97.77	88.01	96.80	71.69	92.50	94.99	93.69	96.13	89.38	93.92
Discard 1536	93.44	88.50	96.99	86.89	96.25	72.00	92.94	94.80	92.04	94.69	88.97	94.12

The best results for each metric are highlighted in bold.

**Figure 11:**
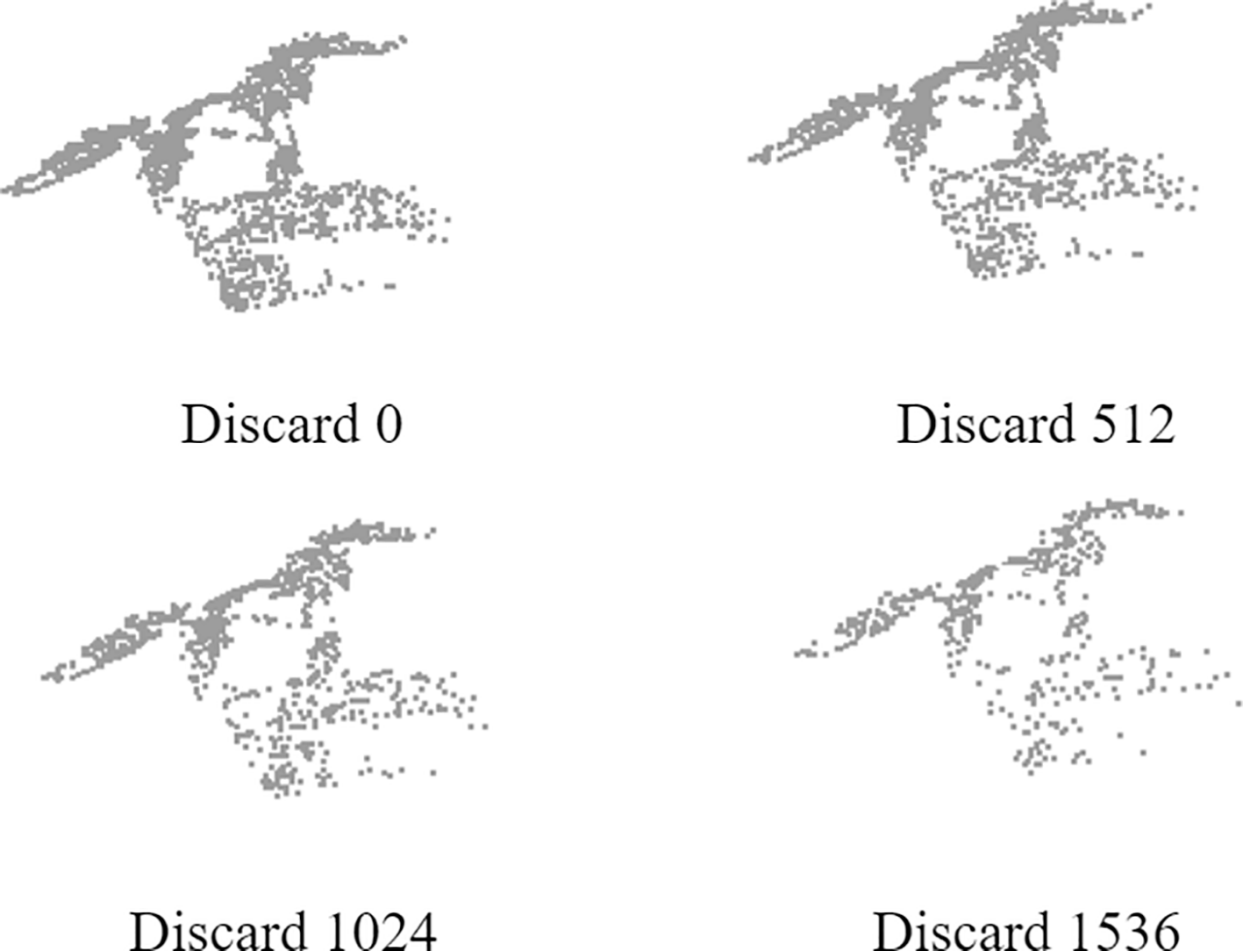
Effect picture of input discard.

As illustrated in **Figure 11**, we demonstrate the visual effects of this density variation by sequentially removing 0, 512, 1,024, and 1,536 points from a sample plant point cloud. This progressive point removal creates four distinct density levels, enabling comprehensive analysis of the model’s segmentation capability across different data completeness scenarios.

With the increase of the number of discarded points, the acc and mIoU of the model show a gradual downward trend.The model achieved peak performance (95.46% acc, 90.41% mIoU) with the complete point cloud (0 points dropped). Performance degradation became progressively more pronounced with increasing point removal: when discarding 512 points, we observed decreases of 1.24 percentage points (94.22% acc) and 1.05 percentage points (89.36% mIoU). Further removal to 1024 points resulted in accumulated reductions of 1.50 (93.96% acc) and 1.61 percentage points (88.80% mIoU) relative to baseline. The most significant performance decline occurred at 1536 points dropped, showing decreases of 2.12 percentage points (93.34% acc) and 2.11 percentage points (88.30% mIoU) compared to the full-density baseline. Overall, the performance of the model slowly decreases as the number of discarded points increases, indicating that the model is robust to the loss of point cloud data.

### 5.8 Noise robustness analysis

Due to constraints imposed by data acquisition devices and environmental factors, the actual point cloud data obtained inevitably contains noise interference. To effectively simulate such real-world noise, this study applies random Gaussian perturbations to point cloud coordinates to establish a benchmark for assessing noise robustness. By setting the standard deviation parameter σ, different noise intensities are simulated: moderate noise (σ=0.01, 0.02) and strong noise (σ=0.04, 0.08). The quantitative analysis results on the Pepper dataset (Planest3D) are shown in **Table 14**.

**Table 14:** Comparison of Pepper dataset (Planest3D) under different noise intensities.

σ	acc	mIoU
0	95.60	97.08
0.01	94.87	97.02
0.02	95.50	96.97
0.04	94.55	96.37
0.08	94.62	96.21

Noise robustness experiments on the Pepper dataset show that: under moderate noise (σ ≤ 0.02), the model performance is stable (acc reaches 95.50% when σ = 0.02, close to the noise-free level); In strong noise conditions, task differentiation emerges—when σ = 0.08, acc rebounds to 94.62% (an increase of 0.07% compared to σ = 0.04), but mIoU continues to decline to 96.21% (a decrease of 0.87% compared to the baseline). This reveals that the model has nonlinear adaptability for classification tasks, while overall segmentation tasks are less affected by noise.

## 6 Conclusions

The experimental results indicate that our Graph Convolutional Attention Synergistic Segmentation Network (GCASSN) fully leverages the advantages of graph convolution in local feature extraction and attention mechanisms in global context modelling. In the segmentation tasks on the Plant3D and Phone4D datasets, GCASSN achieves outstanding performance. Compared with classical algorithms (e.g., PointNet, PointNet++, DGCNN, PCT, and Point Transformer), GCASSN achieves an average accuracy of up to 95.46% and an average intersection-to-union ratio (IoU) of 90.41%, while guaranteeing a better time efficiency and low parameter consumption. In cross-species generalization validation, GCASSN outperforms all baseline models across metrics, indicating that the integration of graph convolutional networks and attention mechanisms effectively balances the capture of local geometric details and global structural information. Furthermore, without parameter fine-tuning, GCASSN achieves a mean mIoU of 85.47% and mcIoU of 82.9% on the ShapeNet dataset, demonstrating notable generalization capabilities.

Our experiments also reveal that graph convolution inherently aligns with 3D point cloud processing, as raw 3D point clouds can be naturally represented as graphs composed of unstructured point sets. Therefore, refining edge construction strategies (e.g., dynamic k-nearest neighbors) or optimizing feature aggregation mechanisms could further enhance model performance. The incorporation of Laplacian transformation effectively strengthens feature correlations within point clouds, improving segmentation accuracy. These results suggest that mathematical tools such as Laplacian transformation hold significant potential for feature enhancement and could be extended to other modules for broader performance gains. Validation in input-limited (sparse, noisy) scenarios shows that GCASSN performs well, but there is still room for improvement. Future work could focus on optimizing graph construction strategies or reducing the sensitivity of attention mechanisms to outliers to further improve model robustness. Additionally, the fusion of graph convolution and attention mechanisms remains an open area for exploration. Future research could further investigate deep integration strategies between these two paradigms.

Given that plants follow specific growth patterns, the introduction of time-series models such as RNN or LSTM to model and analyze their growth dynamics has played a crucial role. Future research can explore the deep integration of our GCASSN model with temporal modeling techniques to construct a unified spatiotemporal analysis framework. This fusion is expected to provide new powerful paradigms for research fields such as plant phenotype analysis and precise quantification of growth processes, thereby contributing more insightful technical support and solutions to the development of plant science.

## Data Availability

The original contributions presented in the study are included in the article/supplementary material. Further inquiries can be directed to the corresponding author/s.
